# The United Kingdom Field Epidemiology Training Programme: meeting programme objectives

**DOI:** 10.2807/1560-7917.ES.2019.24.36.1900013

**Published:** 2019-09-05

**Authors:** Paola Dey, Jeremy Brown, John Sandars, Yvonne Young, Ruth Ruggles, Samantha Bracebridge

**Affiliations:** 1 Faculty of Health, Social Care and Medicine, Edge Hill University, Lancashire, United Kingdom; 2 South London Health Protection Team, Public Health England, London, United Kingdom; 3 Health Protection and Medical Directorate, Public Health England, London, United Kingdom; 4 Formerly Health Protection and Medical Directorate, Public Health England, London, United Kingdom

**Keywords:** education, public health professional, programme evaluation, disease outbreaks, epidemiology, public health surveillance, qualitative research

## Abstract

**Introduction:**

Most evaluations of field epidemiology training programmes (FETP) are limited to process measures, but stakeholders may need evidence of impact.

**Objective:**

To ascertain if the United Kingdom (UK) FETP met its objectives to: (i) strengthen capacity and provision of national epidemiology services, (ii) develop a network of highly skilled field epidemiologists with a shared sense of purpose working to common standards and (iii) raise the profile of field epidemiology through embedding it into everyday health protection practice.

**Methods:**

The evaluation consisted of: (i) focus groups with training site staff, (ii) individual interviews with stakeholders and (iii) an online survey of FETP fellows and graduates. Findings were synthesised and triangulated across the three evaluation components to identify cross-cutting themes and subthemes.

**Findings:**

Eight focus groups were undertaken with 38 staff, ten stakeholders were interviewed and 28 (76%) graduates and fellows responded to the survey. Three themes emerged: confidence, application and rigour. FETP was perceived to have contributed to the development, directly and indirectly, of a skilled workforce in field epidemiology, increasing stakeholders’ confidence in the service. Graduates applied their learning in practice, collaborating with a wide range of disciplines. Fellows and graduates demonstrated rigour by introducing innovations, supporting service improvements and helping supervisors maintain their skills and share good practice.

**Conclusion:**

The UK FETP appears to have met its three key objectives, and also had wider organisational impact. FETPs should systematically and prospectively collect information on how they have influenced changes to field epidemiology practice.

## Introduction

Field Epidemiology Training Programmes (FETPs) contribute to the development of national and global health security infrastructure [1]. For example, during the 2014 Ebola outbreak, the need for trained field epidemiologists was evident internationally; while there was international and regional capacity, there was reportedly a lack of local field epidemiologists to curtail the epidemic [2,3]. In England, a 2008 review of health protection epidemiology identified the need for “*greater professional esprit de corps among epidemiologists*” and recommended the development of a field epidemiology programme to recruit and retain epidemiologists who are “*fit for purpose”* [4].

Within the published literature, there are several international studies reporting the experiences and lessons learnt while setting up FETPs [5-7]. Others report evaluations of processes, such as the number of fellows trained, activities undertaken and/or graduate outcomes, e.g. perceived confidence in key aspects of practice, number of papers published and/or graduate career goals/trajectories [8-16]. Some of these have been used as indicators to monitor the progress of different FETPs [17]. However, there is limited information on FETP impact: Lopez et al. provide a few examples of when policy or services have changed due to work undertaken by trainees [10] and Andre et al. report an improvement in timeliness of surveillance reporting in Benin as a consequence of short-term training of local public health staff [18], but these are not undertaken from the perspective of the service’s stakeholders or across the range of field epidemiology practice.

### Objectives and set up of the programme

The United Kingdom (UK) FETP is an internationally accredited two-year fellowship programme established in 2011. The programme’s objectives were set by the Health Protection Agency, the predecessor to the current national public health agency, Public Health England (PHE). Its objectives are to: (i) strengthen capacity and provision of national epidemiology services, (ii) develop a network of highly skilled field epidemiologists with a shared sense of purpose working to common standards and (iii) raise the profile of field epidemiology by embedding it into everyday health protection practice. In line with other national FETPs [7], it comprises face-to-face training modules that provide knowledge and skills. The focus is on developing expertise in both infectious disease and environmental epidemiology, using a mostly ‘learning-by-doing’ approach. Each fellow is assigned to a recognised training site and works towards achieving defined competences with a dedicated supervisor; fellows’ outputs and the training site are independently and systematically monitored by the FETP Director and FETP Advisory Group. Currently, the programme is delivered collaboratively with the European Programme for Intervention Epidemiology Training Programme (EPIET).

As the programme is now firmly established, with five cohorts that have completed the training, an evaluation was undertaken to ascertain whether it has met the expected objectives, as perceived by FETP fellows and graduates, field epidemiology staff within recognised training sites and key stakeholders.

## Methods

The framework for the evaluation was Kirkpatrick’s model for evaluating training [19-21]. The study was focused on levels 3 and 4 of this model. Level 3 is about behaviour, that is, “*the degree to which participants apply what they learned during training when they are back on the job”,* and level 4 is about results, that is, “*the degree to which targeted outcomes occur as a result of the training and the support and accountability package”* [22]. The study was conducted by academic researchers commissioned by PHE, with assistance from an FETP project team comprising the then FETP Director and two members of PHE’s FETP Advisory Group. There were three study components: (i) focus groups undertaken with supervisors and staff of recognised training sites; (ii) individual interviews conducted with stakeholders identified by the FETP project team as either senior policymakers/managers, senior health protection professionals or international experts in the field; and (iii) an online survey of graduates and current fellows (Supplementary material S1). The questions in each study component were designed to elucidate the different perspectives of each group and, therefore, provide a more in-depth and rounded understanding.

### Information gathering

The focus groups, individual interviews and online questionnaires were completed between January and March 2018. They were conducted by academic researchers not involved in the programme, in order to reduce bias and help facilitate more open responses to the questions. The semi-structured focus group interviews, lasting 45–60 minutes, were undertaken with each team either face-to-face at the training sites or via an Internet interface. Training site supervisors were asked to identify the staff they felt should participate in the interviews, which might include other epidemiologists, health protection professionals, information scientists and/or administrative staff. These interviews concentrated on the impact of hosting FETP fellows (see Box). The individual semi-structured telephone interviews with key stakeholders were completed in 15–20 minutes and focused on the FETP’s contribution to UK field epidemiology’s capacity and capability (see Box). In both group and individual interviews, participants provided illustrative examples of the impact of the FETP on their areas of practice. All interviews were digitally recorded. The online questionnaire was delivered to both current fellows and graduates. The first part of the survey was about the participants’ preparedness and involvement in key areas of field epidemiology and the second part was about the FETP’s impact on careers and benefit to organisations. Given the emphasis of the second part, only graduates completed both parts of the survey. The survey was distributed via email, with a reminder 3 weeks later.

Box **Interview schedules, United Kingdom Field Epidemiology Training Programme evaluation, 2018**

**A. Training site interview schedule**

The fellow’s contribution to:

• management of acute problems such as outbreaks, environmental hazards and incidents;

• development and management of surveillance systems;

• development of standards and quality assurance;

• writing of reports and policy documents;

• initiating, implementing and disseminating research;

• training and mentorship;

• team development and

• development of networks and partnerships.

The resources:

• gained from being part of the EPIET-associated programme,

• needed to support the fellow and

• gained from hosting the fellow.

Indirect benefits to the team and individual team members, such as:

• new knowledge and skill acquisition,

• improved work quality,

• higher confidence in undertaking work,

• a raised profile,

• continued professional development,

• career development and

• greater academic output.

**B. Individual stakeholder interview schedule**

• How aware are you of the FETP/its fellows in the UK?

• How has the technical quality, capacity and capability of field epidemiology and surveillance changed over time?

• How has the FETP contributed to changes in the technical quality, capability and capacity of field epidemiology services nationally?

• Has the FETP had any impact outside of PHE, e.g. in academia or internationally?

• What further aspects of UK field epidemiology/health protection could the FETP contribute to?

EPIET: European Programme for Intervention Epidemiology Training; FETP: Field Epidemiology Training Programme; PHE: Public Health England; UK: United Kingdom.

All potential participants were briefed by the FETP Director, then advised that an academic researcher (PD) would be in contact. They were told they could opt out of the study before their contact details were passed on to the researcher by informing the FETP Director. Alternatively, they could opt out by informing the researcher directly. Written consent was obtained from participants before interviews commenced; consent was implied when questionnaires were completed.

### Data analysis

The data were analysed using an interpretative approach that combined quantitative and qualitative aspects of data collection to provide factual and representative data. Transcripts were analysed using thematic framework analysis [23]. First, one researcher read each transcript several times before establishing a thematic framework for each data collection component, guided by the interview questions, and adding any new themes and subthemes not covered by the interview questions. A second researcher then independently read some of the transcripts to check the thematic framework suggested by the first researcher. The first researcher then discussed the thematic framework agreed upon with the second researcher with the FETP project team to substantiate the context. Coding took place, whereby key illustrative quotations for themes and subthemes contained within the framework were identified from within the collected data. Quantitative survey data were descriptively analysed. Findings were synthesised across the three data collection components to identify cross-cutting themes and subthemes. Findings were integrated across all components using triangulation [24], whereby the analysis of each component was compared with the identified cross-cutting themes and subthemes.

The researchers undertook the analysis and the FETP Advisory Group members only had access to aggregate data that supported the interpretation of the findings. For the purposes of clarity, in presenting the results we use the term ‘graduates’ when referring to survey respondents who have exited the programme and ‘fellows’ for those who are currently in the programme.

### Ethics and governance approval

This study was approved by the Chair of Edge Hill University Faculty of Health and Social Care Ethics Committee (FOH 180) and by Public Health England’s RESEARCH ETHICS AND GOVERNANCE of Public Health Practice GROUP (PHEREGG) - reference R and D 371.

## Results

In total, eight of the nine teams currently training fellows participated in focus groups. This included 38 staff members: 14 consultant epidemiologists, four consultants in health protection/public health, 13 epidemiologists/epidemiological scientists, four information scientists and three administrative staff members. Of the 13 stakeholders approached— including national and international epidemiologists, national policymakers, and national and local health protection leads—10 agreed to participate in individual interviews. After initial contact and follow-up 3 weeks later, 28 of 37 fellows and graduates had responded to the online survey, 19 of whom were graduates. Three cross-cutting themes relating to the objectives of the programme were identified from the three study components’ responses: confidence, application and rigour. Each theme had four subthemes. (Figure).

**Figure:**
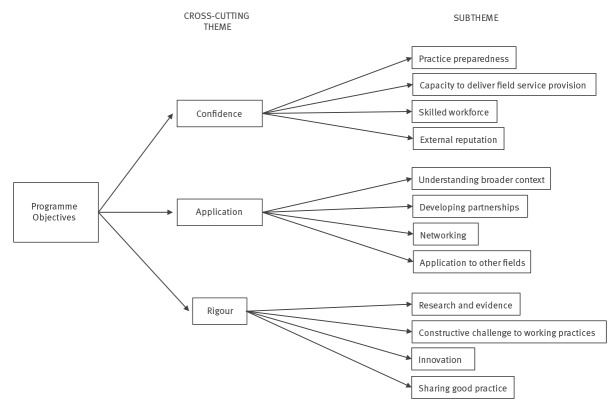
Cross-cutting themes, United Kingdom Field Epidemiology Training Programme evaluation, 2018

### Confidence

While it was acknowledged that recent structural changes to PHE’s delivery of field epidemiology services had a positive impact on these services’ capacity and capability, it was felt that the FETP had also greatly contributed to the development of a skilled workforce. The FETP had a direct impact through training fellows and an indirect impact through developing other staff within local teams and bringing insights from other areas.

“*...I think [the FETP has] enhanced the whole service because of [the teams] having to think about and train [the fellows]…*” Stakeholder 6

“…*the learning, case studies are done in every team now as part of continuing professional development. They were never done before FETP. It’s just seeping into the way of developing staff and not just people who are on FETP.*” Team 4

Graduates and fellows felt confident in applying their learning in field epidemiology work (Table). Teams were confident in graduates’ capability when they returned to the service or took on new roles; graduates were seen to be knowledgeable and able to make decisions, work collaboratively with other disciplines and organisations, and have new and broader perspectives.

**Table:** Number of survey respondents involved in and prepared/somewhat prepared for key areas of epidemiological practice, United Kingdom Field Epidemiology Training Programme evaluation, 2018

Key area of practice	Number of respondents	Number involved	Number prepared/somewhat prepared
Managing acute problems	25	23	24
Managing surveillance systems	25	22	22
Communicating epidemiological information	25	24	22
Providing scientific basis for programme and policy decisions	25	20	18
Developing networks	23	22	20
Raising field epidemiology’s profile	24	23	21

*“I have been able to work more independently and take on responsibility that would have not been considered feasible before FETP.”* Survey respondent

*“…I’d say technical skills, outbreak skills and then the collaborative skills. [I feel] very confident in teaching and training and leading others, and have taken on more senior roles since I came back…very ably.”* Stakeholder 8

Stakeholders attributed their greater confidence in field epidemiology service provision to the influence of the FETP. Teams also felt that fellows helped to raise field epidemiology’s profile locally and internationally, as per the FETP’s objectives, and contributed to increasing stakeholders’ confidence in the benefits of the service provision.

*“…now when we ring them up and say we are having an outbreak meeting this afternoon and we’d like you to contribute, they can always put somebody there…I think part of that is because they’ve all upped their game a little bit by having the input and contribution of the people from the training programme.”* Stakeholder 5

### Application

FETP graduates continued to apply the skills they had developed following completion of the programme (Table) and most (15/16 respondents) strongly agreed or agreed that completing the programme had benefited their employing organisation.

“*I now actually understand what surveillance is and should be for! I’m trying to apply this knowledge to surveillance of physical activity…I wouldn’t have been confident in this had I not done FETP.*” Fellow

Many of the benefits to employers were felt to have resulted from the onsite training, although some felt that further exposure to frontline local health protection activities, beyond field epidemiology, would help fellows understand the complexity of this type of work and facilitate better working relationships.

*“…they’re acquiring the competencies that they need in a real-world service context, rather than some abstract environment where someone is teaching them this, but not necessarily being able to put it into practice.*” Team 7

*“I think something that would benefit [fellows] as part of their training programme is to go and spend a couple of weeks working in the health protection team…[then] they will understand all the things we need and where we are coming from and the kind of constraints and the other workload that people will be trying to manage at the same time as doing an outbreak investigation.*” Stakeholder 6

Through the EPIET programme, graduates and fellows had developed strong networks and there was evidence that this had a direct benefit for the training site teams and could impact population health.

*“…developing that network across Europe or even internationally with fellow epidemiologists is absolutely, you know, like gold dust really. It’s fantastic, and you can call upon the people that you met…the facilitators or even the fellows. Even now, in my work now, as opposed to the training scheme…I do collaborate and ask them questions. It doesn’t matter where they are. So I would say it’s absolutely invaluable.”* Team 1

*“I actually got a tip-off from [a] public health institute…telling us that some samples from [a] producer…[that] we thought was linked to an outbreak in the UK had arrived at the reference laboratory, which meant that we could…get hold of them then and do some sequencing to show that was the source of the outbreak.”* Team 2

Hosting fellows had helped teams further develop partnerships with other disciplines and organisations—e.g. through joint work, networking or delivering learning—and the programme itself was felt by some stakeholders to have promoted national partnerships.

*“…some outbreak training for environmental health officers…was delivered…when we do specific training. I think then the skills transfer.”* Team 2.

*“I think there [are] some other benefits that we get from the FETP. One is it helps cement relationships across the UK between Public Health England and devolved administrations and their public health services.”* Stakeholder 10

There were comments about the potential application of FETP skills training for other fields of epidemiology, disciplines and organisations. Similarly, there were comments about increasing awareness of FETP graduates’ skills among other parts of the employing organisation. This would enhance the benefits to the organisation and the career options for graduates.

*“Given that the non-communicable disease agenda is…rising in terms of scale—and not just in the UK, but globally—the application of field epidemiology-type skills might be equally relevant to non-communicable diseases.”* Stakeholder 7

*“And I think there’s a piece of work [to be done] possibly around awareness of the training programme and what it involves, just outside of perhaps the field epidemiology sphere, because you talk to colleagues and, say, [senior posts within the organisation] don’t really know what it is and what skills are being developed. So…if you’re going for jobs in that room, [FETP training] doesn’t really mean anything.”* Team 1

### Rigour

The added value of fellows working within training sites was strongly evident. Fellows often introduced innovations or skills, or undertook work that led to changes in practice. In particular, teams mentioned statistical tools and techniques, automated reports, and commissioning and decommissioning surveillance systems.

“*I think they can bring sort of a new way of working in, depending on the skills that they bring in and sort of where they’ve been working before. So they can bring ideas in, which is always nice. Because when you’re used to doing something for so long, you do get a new set of eyes coming, going all, you know, ‘You could try it this this way.’*” Team 7

Hosting fellows at training sites could also pose a constructive challenge to working practices, leading to changes in behaviour and culture. This led to increased rigour in approaches, not only because of new techniques, but also because trainers had to refresh their own knowledge to facilitate supervision and teach in the programme.

*“...but, actually, [the fellows] bring that additional rigour that also comes from the supervision—across the supervision on the site, but also from the FETP team—[and they] sort of provide that kind of additional questioning of approaches. I think there’s a real culture of constructive challenge within the programme that really helps some of our projects get off the ground.”* Team 1

*“[Hosting fellows] does help us maintain our own capacity and skills. It does inspire a bit the rest of the team*.” Team 6

Fellows’ capacity to undertake and publish research, and the focus on this as a competence within the programme, was particularly valued.

“*Research and publications tend to be a bit of an add-on for us. We do and we’re meant to do [such work], but it doesn’t take priority. Whereas, with the fellow...it’s almost like they get protected time to do that and that enables [a] kind of more in-depth work to be done, which is quite important for our stakeholders as well.*” Team 5

There was acknowledgement that hosting fellows had led to sharing new ways of working and developing common standards. In part, this was facilitated by the networking opportunities afforded to site supervisors.

“*I think [the FETP has] helped us develop ways of doing things [that] are…a bit more robust or at least more consistent and generally accepted as a way of doing things. So we’ve got that commonality in approach, which I think’s been quite good for the service overall, nationally.”* Team 4

*“I think [the FETP is] really one of the reasons why field epidemiology service as a whole is quite coherent. We’ve built up…relationships between us and it seems less competitive because we’ve [become] used to working with [other field epidemiology teams] on courses that are FETP.*” Team 5

## Discussion

This evaluation, based on predominantly qualitative approaches, suggests that the UK FETP is perceived to have met expectations by addressing the key objectives. The programme was seen to have strengthened capacity and provision of epidemiology services through developing the confidence of graduates and fellows’ in the application of key areas of epidemiological practice, and through instilling more rigorous approaches in field epidemiology practice. All groups were confident that the FETP had contributed to the key objective of developing a network of highly skilled field epidemiologists with a shared sense of purpose working to common standards. The perception was that these impacts have followed not only from training new staff, but also indirectly from changing behaviours and maintaining skills within the wider field epidemiology workforce, driving adoption of innovations and service improvements, and facilitating networking. Dick et al. highlighted that FETP mentors observed similar benefits, such as increasing host site capacity and enhancing their own skills and opportunities, in open comments on an evaluation survey of the career development of FETP trainees in the United States [8]. Further evidence that the final objective had been met included greater visibility of field epidemiology services within everyday practice and external confidence in the service. Teams felt that their profiles were raised through the publication of rigorous research. However, there was felt to be greater potential for more practical exposure to a broader range of health protection activities during training, which could further embed field epidemiology within wider public health practice. Opportunities were identified for extending the remit of the programme to non-communicable disease, which could have further benefits for the employing organisation and population health. Some other national and regional FETP programmes have tracks in other public health areas, e.g. cancer screening and non-infectious disease surveillance [7,25].

The study framework, Kirkpatrick’s model for evaluating training, was principally designed for industry, but is often used to frame evaluations of health professional training [19-21]. FETP evaluations do not often report on behaviour or results (levels 3 and 4 of the Kirkpatrick model), which is consistent with other areas of health training, where organisational, stakeholder and population benefits are less frequently assessed than impacts on learners [26,27]. Yet FETPs are costly and resource intensive, and a lack of evidence of the wider benefits of these programmes could lead to a lack of ongoing financial support [28]. Evaluations focused on organisational and population outcomes are more difficult to undertake. In part, this may be because it can be difficult to directly measure or quantify the attributable benefits. This may be why some evaluations have focused on career trajectories as a proxy for organisational impact [8,15,29]. However, while important, particularly to flag organisational accountability issues if graduates are not finding jobs, employment does not necessarily mean that graduates’ learning prepared them for their new posts or was of additional benefit to stakeholders. Our predominantly qualitative evaluation did provide some evidence that graduates were applying their learning and that the learning had benefits for their employers. Volkov et al. attempted to assess FETP trainees’ application of knowledge and skills through an analysis of the quality of abstracts they had submitted to an international conference [30], and Moolenaar and Thacker through the number and topics of publications by FETP fellows and graduates [29]. However, the quality or frequency of research output does not necessarily equate with service, organisational or public health impact, and research is only one aspect of field epidemiology practice. An important finding of our study was evidence of innovation adoptions, service improvements and potential population health impacts across the range of field epidemiology practice, which both those within and outside of the field epidemiology service attributed to the programme. Given the retrospective nature of the evaluation, these observations are subject to recall bias and, hence, may be underrepresented or misrepresented. Furthermore, these impacts were not quantified. Lopez et al. provided some examples of contributions that FETP trainees made to policy, but again the impacts were not quantified [10]. Quantifying the impact of changes in practice attributable to the FETP programme, through regularly and systematically collecting examples from services and stakeholders, may provide more robust evidence of a FETP’s impact. Such examples may be context specific, both within and between countries, because of local priorities and health challenges. It is therefore important for stakeholders to identify what impacts they consider to be relevant.

A criticism of the Kirkpatrick model is that it ignores the need to establish the link between the training programme and an outcome [31]. It may be desirable—but not always feasible—to undertake a randomised controlled trial of a training programme, particularly when cohorts are small and internal contamination—for example, when the control group becomes exposed to the intervention—may occur when impacts are measured at the organisational or population levels. External contamination is also a challenge. This was highlighted by participants in our study who acknowledged that impact could also be attributable to organisational changes occurring over the same period, albeit they still felt the FETP had made an important contribution. Through the interviews, this evaluation was able to provide insights into how and why participants felt that the FETP had contributed to change and, interestingly, the level of graduates’ preparedness mirrored their ongoing involvement in key aspects of practice.

There were other limitations affecting the interpretation of the evaluation. It elicited views and opinions from teams already involved in the programme and from stakeholders identified by the FETP project team, who may have been biased towards more favourable views. The stakeholders were chosen to represent diversity of experiences of the FETP and organisational roles. This contributed to the richness of the dataset, but also resulted in less consistency in themes within this group. Further research may be warranted on the views of a larger sample of stakeholders. The sample sizes of both the survey and the focus groups were limited by the available pool of participants. The survey’s small pool of participants was further reduced by non-response to the survey and missing responses to specific questions. Finally, a further bias may have been introduced because, although the study components were implemented by academic researchers not involved in the programme in order to facilitate open critique, participants and respondents would have been aware that the FETP Director and Advisory Group would receive the data and be involved in the interpretation of the anonymised findings.

In conclusion, the UK FETP appears to have achieved its purpose and substantively contributed to the capacity and quality of national field epidemiology provision. This is the first study to focus on the impacts for stakeholders. However, to provide quantifiable evidence, FETPs need to systematically and prospectively collect data from various sources on what has changed within field epidemiology services and wider public health provision as a result of hosting fellows and employing graduates.

## Supplementary Data

96000 Supplementary material S1
