# Supplementary material for: The United Kingdom Field Epidemiology Training Programme: meeting programme objectives
Source: Euro Surveill. 2019 Sep 5;24(36):1900013. doi: 10.2807/1560-7917.ES.2019.24.36.1900013 (PMC6737827; doi:10.2807/1560-7917.ES.2019.24.36.1900013)
Supplement: Supplementary material S1 [file 1900013_DEY_SupplementaryMaterialS1.pdf]

Supplementary material 1: Online Survey

This supplementary material is hosted by *Eurosurveillance* as supporting information alongside the article The United Kingdom Field Epidemiology Training Programme: meeting programme objectives on behalf of the authors who remain responsible for the accuracy and appropriateness of the content. The same standards for ethics, copyright, attributions and permissions as for the article apply. Supplements are not edited by *Eurosurveillance* and the journal is not responsible for the maintenance of any links or email addresses provided therein

What year did you begin the Fellowship: (years) How would you describe your training site: generalist or specialist or both or other Please state any other types of placements you completed during your Fellowship (you do not need to give us details on where and what this entailed): In the UK: International: What was your motivation for participation in this training programme (free text):

The following questions will focus on different aspects of Field Epidemiology/Health Protection practice. For each question, you will be prompted to tell us how much you are currently involved, how well the Fellowship is preparing you (or has prepared you) for practice and to provide examples of how the Fellowship has changed your practice and of the most useful aspects of the Fellowship for practice:

Managing acute problems (such as outbreaks, environmental hazards, and natural and man-made disasters)

How often are you involved in this aspect?

Never/rarely/occasionally/a moderate amount/a great deal

How well is the/has the Fellowship preparing you/prepared you for this work?

Prepared/Somewhat Prepared/Neutral/Somewhat Unprepared/unprepared

Please provide any examples of how you feel your practice in this area has changed because of the training (free text)

Please provide any examples of the aspects of the training/programme that you feel have helped improve the quality of your work in this area (free text)

Standard setting for technical work

How often are you involved in this aspect?

Never/rarely/occasionally/a moderate amount/a great deal

How well is the/has the Fellowship preparing you/prepared you for this work?

Prepared/Somewhat Prepared/Neutral/Somewhat Unprepared/unprepared

Please provide any examples of how you feel your practice in this area has changed because of the training (free text)

Please provide any examples of the aspects of the training/programme that you feel have helped improve the quality of your work in this area (free text)

Managing surveillance systems   How often are you involved in this aspect?

Never/rarely/occasionally/a moderate amount/a great deal

How well is the/has the Fellowship preparing you/prepared you for this work?

Prepared/Somewhat Prepared/Neutral/Somewhat Unprepared/unprepared

Please provide any examples of how you feel your practice in this area has changed because of the training (free text)

Please provide any examples of the aspects of the training/programme that you feel have helped improve the quality of your work in this area (free text)

Communicating epidemiological information through reports, presentations etc

How often are you involved in this aspect?

Never/rarely/occasionally/a moderate amount/a great deal

How well is the/has the Fellowship preparing you/prepared you for this work?

Prepared/Somewhat Prepared/Neutral/Somewhat Unprepared/unprepared

Please provide any examples of how you feel your practice in this area has changed because of the training (free text)

Please provide any examples of the aspects of the training/programme that you feel have helped improve the quality of your work in this area (free text)

Providing the scientific basis for programme and policy decisions to prevent and control infectious diseases and environmental hazards

How often are you involved in this aspect?

Never/rarely/occasionally/a moderate amount/a great deal

How well is the/has the Fellowship preparing you/prepared you for this work?

Prepared/Somewhat Prepared/Neutral/Somewhat Unprepared/unprepared

Please provide any examples of how you feel your practice in this area has changed because of the training (free text)

Please provide any examples of the aspects of the training/programme that you feel have helped improve the quality of your work in this area (free text)

Developing networks

How often are you involved in this aspect?

Never/rarely/occasionally/a moderate amount/a great deal

How well is the/has the Fellowship preparing you/prepared you for this work?

Prepared/Somewhat Prepared/Neutral/Somewhat Unprepared/unprepared

Please provide any examples of how you feel your practice in this area has changed because of the training (free text)

Please provide any examples of the aspects of the training that you feel have helped improve the quality of your work in this area (free text)

Raising the profile of field epidemiology

How often are you involved in this aspect?

Never/rarely/occasionally/a moderate amount/a great deal

How well is the/has the Fellowship preparing you/prepared you for this work?

Prepared/Somewhat Prepared/Neutral/Somewhat Unprepared/unprepared

Please provide any examples of how you feel your practice in this area has changed because of the training (free text)

Please provide any examples of the aspects of the training that you feel have helped improve the quality of your work in this area (free text)

Graduated Fellows only:

What is your current role: CCDC/epidemiological scientist/senior scientist/consultant epidemiologist/academic/public health consultant/public health specialist/public health trainee/other (please state)________________

How involved are you currently with the Fellowship programme?

Never/rarely/occasionally/a moderate amount/a great deal

If you are involved, what do you contribute to?

Teaching/Supervision/FETP network/mentoring/other (please state)

Are you a member of any other professional networks?

At local level Yes/No

At regional level Yes/No

At National level Yes/No

At International level Yes/No

How much would you agree with the following statements:

‘The FETP helped me achieve my current role’

Strongly agree/agree/neither agree or disagree/disagree/strongly disagree

‘Taking part in the FETP has helped me achieve my career aspirations’

Strongly agree/agree/neither agree or disagree/disagree/strongly disagree

‘I am still using the skills that the FETP trained me for’

Strongly agree/agree/neither agree or disagree/disagree/strongly disagree

‘Taking part in the FETP has been beneficial to the organisations that I work for’

Strongly agree/agree/neither agree or disagree/disagree/strongly disagree

Please give any examples of how the Fellowship has helped you, or not, in achieving your current role and/or career aspirations (free text):

Please give any examples of any other initiatives or training that you feel would help you achieve your career aspirations

Please give any examples of how you feel the Fellowship has been beneficial to the organisations you work for (free text):

Do you have any other comments?
